# Chemokines simultaneously bind SARS-CoV-2 nucleocapsid protein RNA-binding and dimerization domains

**DOI:** 10.1186/s12985-025-02658-1

**Published:** 2025-03-17

**Authors:** Alberto Domingo López-Muñoz, Jonathan W. Yewdell

**Affiliations:** Cellular Biology Section, Laboratory of Viral Diseases, NIAID (NIH), Bethesda, MD USA

## Abstract

Viruses express chemokine (CHK)-binding proteins to interfere with the host CHK network and thereby modulate leukocyte migration. SARS-CoV-2 Nucleocapsid (N) protein binds a subset of human CHKs with high affinity, inhibiting their chemoattractant properties. Here, we report that both N’s RNA-binding and dimerization domains participate individually in CHK binding. CHKs typically possess independent sites for binding glycosaminoglycans (GAG) and their receptor proteins. We show that the interaction with the N protein occurs through the CHK GAG-binding site, pointing the way to developing compounds that block this interaction for potential anti-coronavirus therapeutics.

## Introduction

Chemokines (CHKs) are secreted from cells and immobilized on cell surfaces by interacting with glycosaminoglycans (GAGs) through their GAG-binding domain. CHKs bound to endothelial cells interact through their receptor-binding domain with leukocyte’s cell surface G protein-coupled receptors (GPCRs). Consistent with the important role of CHKs in anti-viral immunity, viruses have evolved CHK-binding proteins (vCBPs) that interfere with the CHK network to modulate leukocyte migration [1]. vCBPs modulate CHK activity by binding the CHK GAG- or receptor-binding domain, or both.

The relevance of vCBPs as viral countermeasures against the host immune response is emphasized by the large number of known inhibitory vCBPs [2, 3]. Myxoma virus M-T7, vaccinia virus A41, ectromelia virus E163, murine gammaherpesvirus-68 M3 and animal alphaherpesviruses gG, are examples of secreted vCBPs that sequester CHKs. By impairing GAG-CHK or CHK-receptor interactions, these vCPBs disrupt chemotactic gradients to inhibit leukocyte infiltration of infected tissues [4].

Human coronavirus (HCoV) N plays critical roles in viral genomic packaging, transcription and immunoregulation [5, 6]. SARS-CoV-2 N contains three major structural domains: N-terminal domain (NTD), central linker, and C-terminal domain (CTD) [7]. The NTD and CTD are RNA-binding sites [8, 9]. N monomers form a stable dimer through their CTD [9].

We reported that the SARS-CoV-2 N protein is abundantly expressed on the surface of both infected and neighboring uninfected cells. Cell surface N enables activation of Fc receptor-bearing immune cells with anti-N antibodies (Abs), and inhibits in vitro leukocyte chemotaxis by binding 11 human CHKs with high affinity [10, 11]. N was identified as the first secreted HCoV vCBP able to bind CHKs. Here, using bio-layer interferometry (BLI), we map CHK binding to defined N protein domains.

## Materials and methods

### CHKs, GAGs and Abs

Recombinant human CHKs used in this study CCL26 (# 300-48), CCL28 (# 300-57), CXCL10 (# 300-12) from PeproTech, were reconstituted in DPBS 0.1% BSA at 10 µM, aliquoted and stored at − 80 °C. Chondroitin sulfate B (# C3788) was obtained from MilliporeSigma, reconstituted in deionized _i_H_2_O at 1 mg/mL, aliquoted and stored at 4 °C. Anti-SARS-CoV-2 N Abs were obtained from GeneTex: Ab 1 (# gtx635686), Ab 2 (# gtx635688), Ab 3 (# gtx635685), Ab 4 (# gtx635679), Ab 5 (# gtx635687), Ab 6 (# gtx635808), Ab 7 (# gtx635689). We confirmed mAb binding to N NTD vs. CTD via dot-blot using recombinant full-length N (aa 1-419, Sino Biological # 40,588-V07E), NTD (aa 49-175, # 40,588-V07E10) and N CTD (aa 248–365, # 40,588-V07E5).

### BLI assays

Binding assays were performed on an Octet Red384 instrument (Sartorius) at 30 °C with shaking at 1,000 rpm, in 96-well (half area, black, flat bottom, polystyrene) microplate (Corning # 3686).

Streptavidin (SA) biosensors (Sartorius # 18-5019) were hydrated for 10 min in running buffer (DPBS (Gibco # 14,190-144), 1% BSA, 0.05% Tween-20). After initial baseline equilibration for 60 s in running buffer, SARS-CoV-2 N protein (2X-StrepTag tagged) in lysis buffer (50 mM tris–HCl (pH 7.4), 150 mM NaCl, 5 mM KCl, 5 mM MgCl2, 1% NP-40, and 1 × protease inhibitors (Roche # 4,693,159,001)) from crude lysates of transfected cells (see details below) were loaded into SA biosensors up to 5 nm binding response for 300 s, followed by baseline equilibration for 90 s in running buffer.

For Ab-competition assays, after N loading and baseline equilibration, hydrated SA biosensors were dipped for 180 s in 100 µL of running buffer containing 0.02 mg/mL of indicated anti-N Ab, followed by baseline equilibration for 90 s in running buffer. Association of each CHK (100 nM) in running buffer at indicated concentration was carried out for 300 s, followed by dissociation for 180 s. The binding response of each CHK to N, in the absence Abs (control), was considered 100% of binding response.

For GAG-competition assays, chemokines (100 nM) were incubated in running buffer alone or with indicated concentrations of soluble GAGs for 10 min at room temperature, before N loading and baseline equilibration. Association of each CHK in running buffer at indicated concentration was carried out for 180 s, followed by dissociation for 180 s. The binding response of each CHK to N, in the absence GAGs, was considered 100% of binding response. The binding response of each CHK to N was obtained from data analysis with Octet Analysis Studio Software, version 12.2.2.26.

### SARS-CoV-2 N protein (2X-Strep) production

Batches of SARS-CoV-2 N 2xStrep in crude lysates were obtained from 30 × 10^6^ HEK293-FT cells transfected with 30 μg of Addgene plasmid # 141,391 (pLVX-EF1alpha-SARS-CoV-2-orf8-2xStrep-IRES-Puro, a gift from Nevan Krogan) with TransIT-LT1 Transfection Reagent (Mirus Bio). After 24 h, transfected cells were selected with puromycin (10 μg/ml; InvivoGen, no. ant-pr-1). After 48 h, transfected cells were trypsinized, washed with DPBS, and lysated for 30 min at 4 °C in 1 ml of lysis buffer (50 mM tris–HCl (pH 7.4), 150 mM NaCl, 5 mM KCl, 5 mM MgCl2, 1% NP-40, and 1 × protease inhibitors (Roche # 4,693,159,001)), followed by centrifugation at 1000 g at 4 °C. Clarified supernatants (crude lysates) were collected, aliquoted, stored at − 20 °C, and characterized by immunoblotting using IRDye 680RD Streptavidin (LI-COR # 926-68,079).

HEK293-FT (# CRL-11268) cells were from the American Type Culture Collection (ATCC). Cells were grown in DMEM with GlutaMAX (Thermo Fisher # 10,566,016), supplemented with 8% (v/v) not heat-inactivated fetal bovine serum (HyClone, no. SH30071.03). Cells were cultured at 37 °C with 5% CO2, and passaged at ~ 80 to 90% confluence.

## Results and discussion

### SARS-CoV-2 N uses both the RNA-binding and dimerization domains to bind CHKs

We reported that, among 64 human cytokines examined by BLI, N binds CCL5, CCL11, CCL21, CCL26, CCL28, CXCL4, CXCL9, CXCL10, CXCL11, CXCL12β, and CXCL14 with micromolar to nanomolar affinities [11]. For this study, we selected 3 CHKs (CCL26, CCL28, and CXCL10) and determined their binding to N NTD *vs.* CTD via a BLI Ab-based competition assay.

**Fig. 1:**
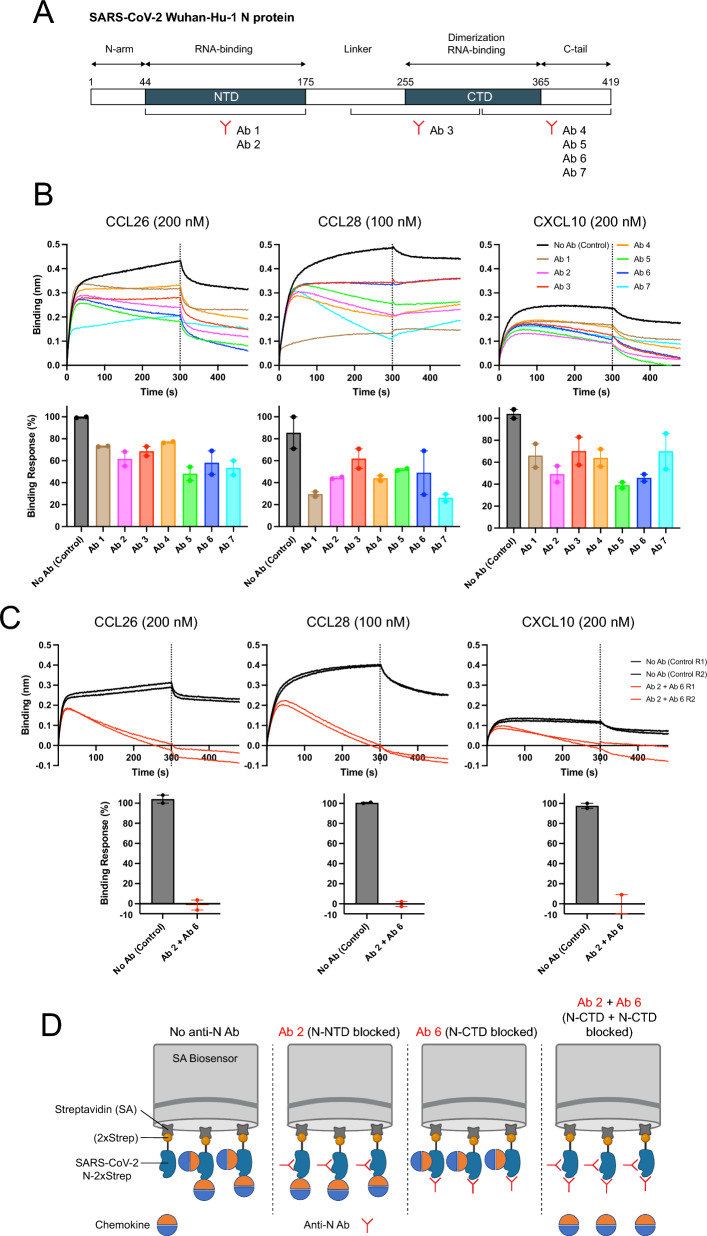
Both the RNA-binding (NTD) and dimerization (CTD) domains of the N protein contribute to CHK binding. **A** Schematic of the N protein domain architecture and binding sites of each anti-N Ab used in this study. **B**, **C** Anti-N Abs bound to immobilized N reduce N CHK-binding activity. BLI sensorgrams of binding assays showing association and dissociation phases of the interaction between the N protein and 3 positively bound CHKs (upper graphs). Prior to the association phase with CHKs, immobilized N was incubated with **B** one of each anti-N Ab, or **C** simultaneously with Ab 2 and Ab 6, up to binding saturation. The dotted line indicates the end of the association phase. The binding response of each CHK to N, in the absence of prior incubation with anti-N Abs, was considered 100% of binding response (lower graphs). Data represent the mean ± SEM (n = 2). One representative assay out of three independent assays performed in duplicate is shown. **D** Proposed model of biomolecular interaction between N and CHKs, where both the NTD and CTD RNA-binding domain of N act as CHK-binding sites

The Ab-competition assay entails immobilizing recombinant N with two copies of StrepTag (N-2xStrep), a streptavidin-binding peptide, to a streptavidin (SA) derivatized biosensor. We then incubate biosensor-bound N with a saturating amount (as determined by a plateauing BLI binding signal) of a mAb specific for N NTD or CTD (Fig. 1A), followed by incubation with one of the selected CHKs, whose association and dissociation is measured via BLI. The binding response to N with each CHK in the absence of Abs (control) is used to establish the 100% binding value.

We observed competition of CHK binding to N ranging from 75 to 25% depending on the mAb and CHK combination queried (Fig. 1B). These results are consistent with the premise that each CHK tested binds to both the N NTD and CTD.

To confirm the contribution of N NTD and CTD to CHK binding, we selected two anti-N mAbs (Ab 2, an anti-N-NTD Ab, and Ab 6, an anti-N-CTD Ab, Fig. 1A) which individually block the CHK binding response of N by roughly 50% (Fig. 1B). We then incubated biosensor-bound N with saturating amounts of the mAb mixture, followed by incubation with one of the three selected CHKs. Simultaneously blocking N NTD and CTD completely abrogated binding of CCL26, CCL28 and CXCL10 (Fig. 1C).

Together, these findings indicate that both the RNA-binding and dimerization domains of the N protein independently bind CHKs (Fig. 1D).

### SARS-CoV-2 N bind CHKs through their GAG-binding site

**Fig. 2:**
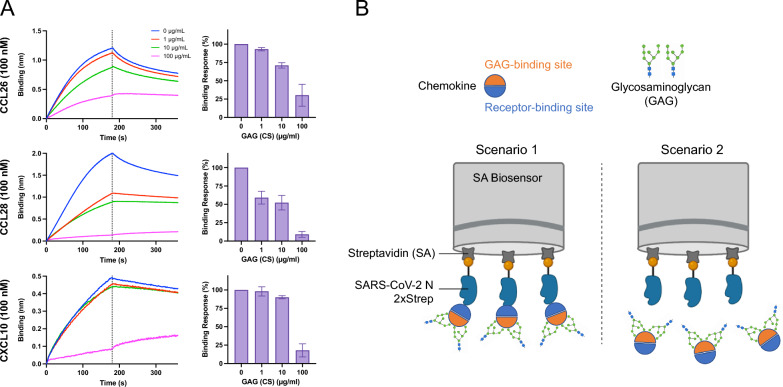
CHK interaction with the N protein occurs through the GAG-binding site of CHKs. **A** BLI sensorgrams of binding assays showing association and dissociation phases of the interaction between the N protein and CHKs, in the presence of GAG (left graphs). Prior to the association phase, CHKs were incubated with increasing concentrations of chondroitin sulfate (CS). The dotted line indicates the end of the association phase. The binding response of each CHK to N, in the absence of GAG (CS), was considered 100% of binding response (right graphs). Data represent the mean ± SEM of three independent experiments. The N protein does not bind CS [11]. **B** Proposed scenarios of CHK-N biomolecular interaction, where CHKs might bind to N through their receptor-binding site (scenario 1), or through their GAG-binding site (scenario 2). Prior incubation of CHKs with soluble GAGs blocks the GAG-binding site of CHKs, i.e., the CHK availability for interaction through its GAG-binding site

CHKs have independent binding sites for GAGs and CHK receptors (i.e., GPCRs). To determine their contribution to binding N, we performed GAG-competition assays by BLI. We pre-incubated CCL26, CCL28, and CXCL10 with chondroitin sulfate (CS), a soluble GAG we previously reported not to detectably bind to N [11]. We incubated each of the CHKs used above with CS and examined the effect on CHK binding to biosensor-bound N. The binding response obtained from each CHK binding to immobilized N in the absence of CS was considered 100% of binding. This revealed that for each CHK tested, CS blocked CHK binding to N in a dose-dependent manner (Fig. 2A). This is consistent with scenario 2 in Fig. 2B, in which CHKs predominantly interact with N via their GAG-binding domain.

Our results show that via their GAG-binding site, CHKs independently interact with each of the SARS-CoV-2 N RNA-binding and dimerization domains. On average, N binds CHKs with a much higher affinity (nanomolar range) than ssRNA (micromolar range) [8, 9, 11]. Our previous analysis of BLI kinetic data between N and each of the 11 CHK bound by N showed heterogeneous binding profiles, deviating from a classical 1:1 interaction [11]. For these data the optimal model to calculate the affinity constants was the 2:1 heterogeneous ligand model based on CHK binding at two independent N sites [12], which we confirm in the present study.

Using HADDOCK and AlphaFold2-Multimer software for in silico modeling of interaction, Tomezsko et al*.* reported a high specificity of docking/interaction between CXCL12β and the SARS-CoV-2 N dimerization domain (CTD) [13]. While they also noted contacts between CXCL12β with some residues located in the N RNA-binding domain, the N NTD was not identified as an independent CHK-binding domain. We note that while in silico prediction of protein interaction is rapidly improving, it is not definitive, and that empirical structural studies are required to establish the basis of these interactions more firmly. Even so, it will be interesting in future studies to use BLI to test the Tomezsko et al*.*’s prediction that a number of SARS-CoV-2 N variants that have evolved during the pandemic bind with lower affinity to four CHKs from our previous experimental screening [11], also nine cytokines that we reported as negative binders using BLI but had increased in silico interactions with N variants.

It will also be interesting to compare the structural basis for CHK binding to N encoded by HCoV-OC43 and other human coronaviruses, which we reported to be expressed on the surface of infected and surrounding cells, binding CHKs, and inhibiting CHK-induced chemotaxis [14]. While SARS-CoV-2 and HCoV-OC43 N proteins only share 38% sequence identity, N domain architecture and overall structure is highly conserved across HCoVs [6, 15].

## Data Availability

All data are provided within the manuscript and figures.
